# HMGB1 in inflammation and cancer

**DOI:** 10.1186/s13045-020-00950-x

**Published:** 2020-08-24

**Authors:** Shumin Wang, Yi Zhang

**Affiliations:** 1 Biotherapy Center & Cancer Center, The First Affiliated Hospital of Zhengzhou University, Zhengzhou, 450052 China; 2 State Key Laboratory of Esophageal Cancer Prevention & Treatment, Zhengzhou University, Zhengzhou, 450052 China; 3 Henan Key Laboratory for Tumor Immunology and Biotherapy, Zhengzhou, 450052 China

**Keywords:** HMGB1, RAGE, TLR, DAMP, Inflammation, Cancer

## Abstract

High mobility group box 1 (HMGB1) is a non-histone chromatin-associated protein widely distributed in eukaryotic cells and is involved in DNA damage repair and genomic stability maintenance. In response to stimulus like bacteria or chemoradiotherapy, HMGB1 can translocate to extracellular context as a danger alarmin, activate the immune response, and participate in the regulation of inflammation and cancer progression.

High mobility group box 1 (HMGB1) is a highly conservative nucleoprotein and belongs to the group of non-histone chromatin-associated protein. It was first extracted from calf-thymus chromatin in 1973 and named for its high mobility in gel electrophoresis [1]. Subsequent investigations found that HMGB1 could translocate from the nucleus to the cytoplasm after posttranslational modifications, including acetylation, phosphorylation, and methylation. HMGB1 can be expressed at the neuron membrane as well. In response to chemoradiotherapy or hypoxia, HMGB1 could be transferred to the extracellular context mainly through two ways: active secretion from immunocompetent cells or passive release from apoptotic or necrotic cells. Extracellular HMGB1 transmits danger signals to surrounding cells by interacting with its classical receptors, such as the receptor for advanced glycation end products (RAGE) and Toll-like receptors 2/4/9 (TLR-2/4/9) [2]. In-depth studies implicated that HMGB1 was a multifunctional protein involved in a variety of cellular biological properties, depending on its subcellular localization, post-transcriptional modification, and binding receptors (Fig. 1).

**Fig. 1:**
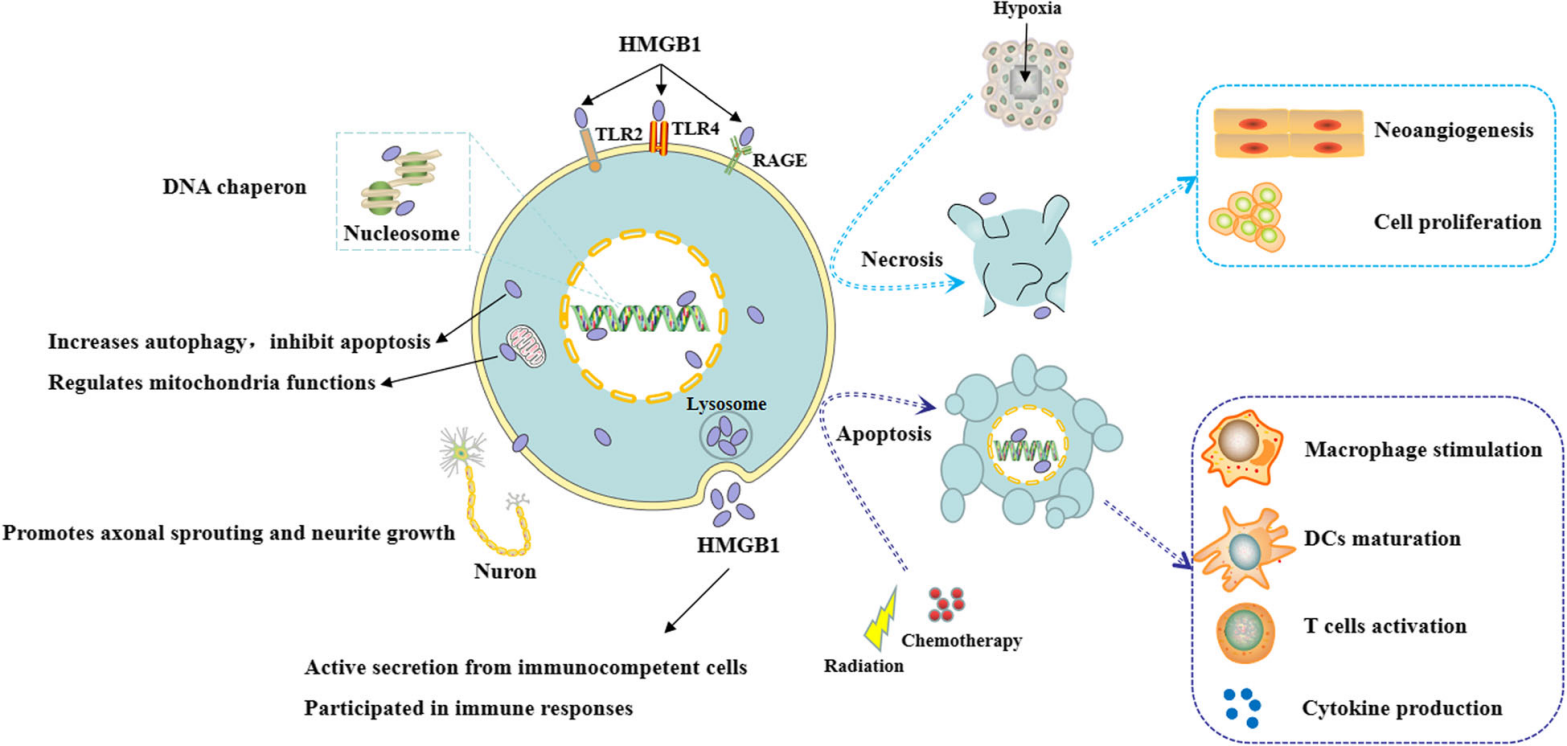
The multi-functions of HMGB1. HMGB1 in the nucleus regulates DNA damage repair and genome stability as a non-histone chromatin-associated protein and DNA chaperon. In the cytoplasm or mitochondria, HMGB1 increases autophagy, inhibits apoptosis, and regulates mitochondria functions. At the membrane, HMGB1 promotes axonal sprouting and neurite growth. HMGB1 can be transferred to extracellular context by two ways: active secretion from immunocompetent cells or passive release from apoptotic or necrotic cells, participating in immune responses

In the nucleus, HMGB1 plays a key role in the process of DNA replication, transcription, chromatin remodeling, and V(D)J recombination, thus regulating DNA damage repair and the maintenance of genome stability as a DNA chaperone. Cytoplasmic HMGB1 is involved in immune responses by increasing autophagy, inhibiting apoptosis, and regulating mitochondrial function [3]. At the membrane, HMGB1 promotes axonal sprouting and neurite growth, activates platelets, and induces cell migration. As a typical damage associated molecular pattern (DAMP), extracellular HMGB1 is involved in many immune responses by promoting immune cell maturation, activation and cytokine production [4]. Extracellular HMGB1 can also interact with chemokines such as CXCL11 to enhance immune responses [5]. As a multifunctional protein, HMGB1 exerts different biological effects under different stimuli. The deregulation of HMGB1 is associated with many diseases, especially inflammatory disorders and cancer.

## HMGB1 in inflammation

HMGB1 plays a critical role in the regulation of both innate and adaptive immune responses to promote immune response to sterile or infectious stimulus [6]. In sterile inflammation during ischemia-reperfusion injury (IRI), HMGB1 becomes disulfide-bonded to increase macrophage production of pro-inflammation cytokines in a TLR-4 dependent manner, indicating that disulfide-bonded HMGB1 can be identified as a diagnosis and treatment biomarker for IRI. When initially secreted by innate immune cells like macrophages, HMGB1 is pro-inflammatory during the early stages of sepsis. However, the extracellular HMGB1 could also induce immune tolerance and immunosuppression when released by other somatic cells. In contrast, intracellular HMGB1 can induce protective autophagy and contribute to cell survival. By delivering lipopolysaccharide (LPS) and promoting endocytosis, HMGB1 activates the noncanonical inflammasome pathway and induces pyroptosis [7]. Pyroptotic macrophage death may accelerate undesirable immune hyperactivity and immunosuppression, which is a potential mechanism associated with late mortality from sepsis [8]. In hepatic infectious disease, the release and activity of HMGB1 as a cytokine could be suppressed by glycyrrhizinic acid (GA) [9]. By binding with TLR4, HMGB1 regulates the hepatitis viruses-induced immunological axis, providing a new therapy strategy for the treatment of acute viral hepatitis in the clinical practice [10].

In severe pulmonary inflammatory diseases including COVID-19, HMGB1 can be secreted in abundance by necrotic pulmonary epithelial cells and innate immune cells. Disulfide-HMGB1 triggers pro-inflammatory cytokine release and further exacerbates severe inflammation [11]. Therefore, HMGB1 might be a potential target for the treatment of inflammation.

## The dual effects of HMGB1 in cancer

According to the alterations of the subcellular locations, receptors, and expression levels, HMGB1 is associated with the hallmarks of cancer proposed by Hanahan and Weinberg [12]. HMGB1 appears to play paradoxical roles during the development and therapy of cancer. On the one hand, HMGB1 can contribute to tumorigenesis. Excessive HMGB1 production caused by chronic inflammatory response seems to be associated with tumorigenesis. For example, by combining with RAGE, HMGB1 plays an important role in regulating oval cells activation and inflammation-associated liver carcinogenesis in mice [13]. In established cancers, HMGB1 produced by tumor cells may exacerbate inflammation-related immunosuppression. For instance, previous research indicated that LPS induced the release of pro-inflammatory cytokines such as IL-1β, IL-6, and TNF-α in a HMGB1-dependent manner to improve colon cancer progression [14]. However, the underlying mechanism of HMGB1 in the transformation of inflammation and cancer needs to be further studied. It has been reported that HMGB1 can be released to extracellular context by necrotic cells under hypoxia in growing solid tumor. Extracellular HMGB1 promotes the release of cytokines such as IL-6 and IL-8 by activating MAPK- and MyD88-dependent NF-κB pathways, which in turn stimulates tumor cells proliferation, angiogenesis, EMT, invasion, and metastasis. Nucleus and cytoplasmic HMGB1 promotes autophagy and inhibits apoptosis of tumor cells to induce chemotherapy resistance [15]. On the other hand, HMGB1 plays a protective role in the suppression of tumor and tumor chemoradiotherapy and immunotherapy. Nucleus HMGB1 assists in the regulation of telomere and maintenance of genome stability. Loss of HMGB1 results in the instability of genome and leads to tumorigenesis. Thus, the roles of HMGB1 in regulation of DNA damage repair and cancer etiology indicate that targeting chromosomal architectural HMGB1 may provide a new perspective for cancer therapy [16]. HMGB1 located in the cytosol or mitochondria may bind to autophagy associated genes like Beclin 1 to regulate cell autophagy and mitophagy. Absence of HMGB1 results in autophagy deficiency and increased apoptosis, leading to tumorigenesis. Intracellular HMGB1 functions as a tumor suppressor by binding tumor suppressor proteins like Rb. But it remains to be studied whether HMGB1 interacts with other tumor suppressors or oncoproteins. Extracellular HMGB1 enhances chemotherapy efficacy by transforming tumor cells from apoptosis to senescence [15]. In addition, HMGB1 can mediate immunogenic cell death during chemoradiotherapy and enhance anti-tumor immunity. In response to chemotherapy like anthracycline or radiotherapy, HMGB1 can be rapidly released from dead cells as an alarming molecule. Upon release from necrotic cells or secreted by activated macrophages, HMGB1 can recruit inflammatory cells and mediate interactions between NK cells, dendritic cells (DCs), and macrophages. Activated NK cells provide an additional source of HMGB1, which is released into the immunological synapse between NK cells and immature DCs, promoting the maturation of DCs and the induction of Th1 response [17]. In addition, HMGB1 produced from NSCLC cells induced by docetaxel can stimulate T cells for anti-tumor immune response and improve immunotherapy effects like CAR-T cells [5]. Therefore, modulating HMGB1 may provide a potential combination strategy for cancer chemoradiotherapy and immunotherapy.

## Conclusion

HMGB1 is a multifunctional molecule that plays a major role in homeostasis as DAMP molecule. HMGB1 plays the complex roles in various biological processes and is involved in the development of many diseases such as autoimmune diseases and cancers. HMGB1-targeted agents including antibodies and inhibitors have shown beneficial results in preclinical inflammatory models such as sepsis. These agents await clinical development.

## Data Availability

Not applicable.

## Abbreviations

HMGB1: High mobility group box 1
RAGE: The receptor for advanced glycation end products
TLR: Toll-like receptors
DAMP: Damage associated molecular pattern
IRI: Ischemia-reperfusion injury
LPS: Lipopolysaccharide
GA: Glycyrrhizinic acid
COVID-19: Coronavirus disease-19
MAPK: Mitogen-activated protein kinase
MyD88: Myeloid differential protein-88
NF-κB: Nuclear factor kappa-light-chain-enhancer of activated B cells
EMT: Epithelial-mesenchymal transition
DCs: Dendritic cells
